# The Economic Implications of Relapse Among Children Recovered from Severe Acute Malnutrition: Results from a Multicountry Prospective Study in Mali, Somalia, and South Sudan

**DOI:** 10.1016/j.cdnut.2025.107616

**Published:** 2025-12-10

**Authors:** Chloe Puett, Sarah King, Sherifath Mama Chabi, Feysal Abdisalan Mohamud, Nancy Lamwaka, Heather Stobaugh

**Affiliations:** 1 Department of Family, Population & Preventive Medicine, Program in Public Health, Health Sciences Center, Stony Brook University, New York, NY, United States; 2 Division of Global Health Protection, US Centers for Disease Control and Prevention, Atlanta, GA, United States; 3 Acción Contra el Hambre, Nutrition Research Department, Bamako, Mali; 4 Action Against Hunger, Nutrition and Health Technical Department, Mogadishu, Somalia; 5 Action Against Hunger, Nutrition and Health Technical Department, Juba, South Sudan; 6 Action Against Hunger, Research and Innovation Department, New York, NY, United States

**Keywords:** severe acute malnutrition, wasting, relapse, cost analysis, economic analysis

## Abstract

**Background:**

The community-based management of acute malnutrition (CMAM) is effective at recovering children from severe acute malnutrition (SAM) and saving lives. However, postdischarge, children may relapse to acute malnutrition (AM), thereby requiring retreatment.

**Objectives:**

This study aims to assess the economic implications of treating children who relapse to AM within 6 mo of recovering from initial treatment for uncomplicated SAM in CMAM programs.

**Methods:**

This study was part of a multicountry prospective cohort study in which children aged 6–47 mo with uncomplicated SAM were followed for 6 mo after recovery in Mali, Somalia, and South Sudan (SSD). Institutional cost data were collected monthly through accounting records or key informant interviews, depending on data availability. Mean costs per treatment episode for each CMAM program component were calculated (initial SAM recovery, relapse to SAM treatment, relapse to moderate AM treatment; 2022 USD).

**Results:**

The cost per child of recovery in initial SAM treatment was $191, $92, and $178 in Mali, Somalia, and SSD, respectively. The cost per child who failed to sustain recovery and required treatment for relapse to SAM was 1.7–2.2 times higher than initial recovery, amounting to an additional $215, $64, and $215/child in Mali, Somalia, and SSD, respectively. Not having to retreat children would save per admitted child on mean $2 in Somalia, $26 in SSD, and $45 in Mali.

**Conclusions:**

This is the first analysis to estimate the cost of retreating children who relapse to AM within 6 mo of recovering from SAM. Our findings show that the cost of treating relapse to SAM is, on mean, twice as high as the initial SAM recovery. Preventing relapse could free up significant resources. Amid reduced global assistance, these results highlight the economic rationale for improving the efficiency of global nutrition programs by reducing relapse rates and strengthening prevention strategies.

## Introduction

Child wasting is a critical global public health concern, with wasted children facing a 3–12 times higher risk of mortality, depending on the severity of their condition, compared with nonwasted children [1]. The community-based management of acute malnutrition (CMAM) is an effective and essential intervention for treating severe acute malnutrition (SAM), forming the backbone of global nutrition efforts aimed at reaching ∼45 million acutely malnourished children at any given time [2].

CMAM provides both outpatient and inpatient care to children with wasting using therapeutic foods and medications tailored to the severity of wasting and the presence of comorbidities. Owing to its demonstrated success in reducing mortality, CMAM has been endorsed by the WHO as the global standard of care, particularly in settings with persistently high rates of wasting. In countries such as South Sudan, Mali, and Somalia, where frequent political instability, food insecurity, and humanitarian crises prevail, wasting prevalence often exceeds WHO emergency thresholds, necessitating ongoing CMAM programming to address the high burden of wasting. Consequently, areas where CMAM services are actively implemented tend to experience lower mortality rates associated with wasting. A recent study conducted across 16 Eastern and Southern African countries found that CMAM programs achieved an mean case fatality rate of just 0.67% [3]. However, a persistent challenge remains: a very wide range of proportions of children relapse to acute malnutrition (AM) after initial SAM recovery. Recent evidence documented ≤63% of children can relapse to AM within 6 mo of recovery, requiring retreatment and additional resources to restore their nutritional health [4].

Reducing the number of repeated episodes of AM would not only improve children’s health but also result in more efficient use of resources. Amid declining humanitarian funding, optimizing the use of available funds has become increasingly urgent [[5], [6], [7]]. Current CMAM programs are resource-intensive interventions that struggle with coverage gaps and annual treatment shortfalls, typically ranging in funding gaps of 53%–65% [8]. Retreatment of relapsed children further strains limited resources, reducing the capacity to reach new children in need and hindering progress toward achieving Sustainable Development Goal Target 2.2. Therefore, it is economically imperative to assess how much money is spent on retreating children to design cost-efficient interventions to reduce post-SAM relapse.

This study is the first known attempt to investigate the economic implications of relapse to AM after recovery from SAM through CMAM programs. The findings are expected to raise awareness within the global nutrition community about the costs of retreating children who have relapsed, while advocating for robust post-SAM relapse prevention efforts.

## Methods

### Study design

This study, conducted within a larger multicountry prospective cohort study on relapse in young children after treatment and recovery for uncomplicated SAM, aimed to evaluate the cost implications of relapse within CMAM programs. The study assessed the financial implications of treating children who relapsed to AM within 6 mo of recovery from an initial SAM episode. A cost-efficiency analysis approach was taken, which assessed the operating costs incurred to achieve a program output, in this case, a child treated [9]. The cohort study followed children aged 6–47 mo for 6 mo postdischarge from CMAM in Mali, Somalia, and South Sudan. Detailed study protocols for both the main cohort study and this substudy have been previously published [10,11]. Ethical approval for this parent study was provided by Solutions Institutional Review Board (reference number #20200310), London School of Hygiene & Tropical Medicine's Research Ethics Committee (#18059), Ministry of Health (MoH) Somalia (MOH&HS/DGO/0429/03/202), the Université Des Sciences, Des Techniques Et Des Technologies De Bamako (2020/202/CE/FMOS/FAPH) in Mali, and the MoH of South Sudan (MOH/ERB6/2020). No additional ethical approval was sought for the economic substudy described here.

### Study setting and programmatic context

The study was conducted at 16 CMAM sites in Mali (9 sites), Somalia (1 site), and South Sudan (6 sites). Sites were selected in regions with well-established CMAM programs and persistently high rates of AM. The sites in South Sudan and Mali were in rural areas, serving nondisplaced populations, whereas the Somalia site was urban, primarily serving internally displaced persons (IDPs). During the study, the study regions in South Sudan and Somalia experienced high rates of AM, frequently reaching Integrated Phase Classification for AM Phase 4 thresholds [12].

The 3 programs each comprised the 4 standard CMAM service components: *1*) inpatient treatment in stabilization centers (SC) for SAM with medical complications; *2*) outpatient therapeutic programs (OTP) for uncomplicated SAM using ready-to-use therapeutic foods (RUTF) and medical care; *3*) management of moderate AM (MAM) in targeted supplementary feeding programs (TSFP) where children receive ready-to-use supplementary foods (RUSF) or fortified blended foods; and *4*) community outreach services.

Although all 3 countries implemented all CMAM service components, there were slight variations in their execution, reflecting variations in each country’s national CMAM protocol (Supplemental Materials, Supplemental Table 1 and Supplemental Figure 1). In Somalia and South Sudan, uncomplicated SAM cases began treatment in OTP until reaching MAM status, at which point they were transferred to TSFP to complete treatment. In Mali, children with SAM received the full treatment in OTP without transfer, resulting in more frequent follow-up visits and receiving only RUTF until discharge. In Somalia and South Sudan, children received both RUTF and RUSF, with follow-up visits moving from weekly to biweekly after transfer from OTP to TSFP service components. OTP and TSFP programs were offered at the same site in all countries, so transfers between program components did not require a location change. The protocols associated with inpatient treatment, MAM management, and community outreach were similar in all 3 countries.

The primary service implementer also differed between countries: South Sudan’s program was entirely run by a nongovernmental organization (NGO), Mali’s program was solely administered by the MoH, and in Somalia, the MoH and an NGO jointly implemented the program. The site in the IDP camp in Somalia had the highest caseload, followed secondly by those in South Sudan, and lastly by Mali’s sites, observing the smallest caseloads overall.

### Data collection

In the parent study, enrolled children were monitored for AM on a monthly basis for 6 mo directly after initial SAM recovery to determine both the cumulative incidence and the point prevalence of relapse to AM. Children found to be malnourished during follow-up were referred for retreatment and continued to be observed until the 6-mo follow-up was completed. Treatment and monthly programmatic caseload data were collected for SCs, OTPs, and TSFPs to track caseloads and the total number of children per service type for both initial SAM treatment and any subsequent relapse treatment, whether for SAM or MAM. Programmatic data were collected for all children’s initial treatment; however, data were not collected for any portion of relapse treatment extending beyond a child’s 6-mo follow-up. For example, if a relapse occurred at month 5, only data for the remaining 1 mo were collected, even if treatment was still ongoing. For these cases, we used the mean length of stay for those children who completed their full retreatment within the 6 mo and applied it to all children who relapsed. This assumed that the length of stay was the same regardless of when within the 6-mo period the case of relapse occurred. Individual treatment data were not collected for children outside the parent study, though they were included in facility-level caseloads.

For study participants, treatment data for initial and relapse treatment included child’s anthropometry [height, weight, and mid-upper arm circumference (MUAC)] at each treatment visit and follow-up visit, length of stay, type and quantity of food commodities used, and visit dates (Supplemental Materials, Supplemental Table 1 and Supplemental Figure 1). AM was defined following each country’s CMAM protocol. For children in Mali and South Sudan, it was defined by either a low MUAC (< 125 mm) or a low weight-for-height *z*-score (WHZ < −2SD), whereas in Somalia, it was defined as AM by low MUAC alone. Across all countries, the presence of edema classified a child as severely malnourished. In Mali, children were discharged as recovered if both their MUAC and WHZ met exit criteria, aligning with the 2023 WHO’s definition of recovery [13]. In South Sudan, discharge was based on the criterion that was low at the time of admission (if both criteria were low at admission, MUAC was typically prioritized as the indicator for discharge). For Somalia, discharge was based on MUAC. These country-specific definitions determined eligibility for both initial SAM treatment and subsequent relapse treatment, as well as inclusion in monthly program caseloads.

Cost data collection and analysis procedures followed recommended processes outlined in costing guides for CMAM programming [14,15]. Across all country programs, we followed best practices for assessing costs of health programs [16] by collecting the same categories of costs to represent a comprehensive set of resources needed to run each program. Each country implemented very similar programming but delivered by different types of institutions (i.e., Ministries of Health, NGOs). Where available, we used institutional accounting data to quantify the cost of resources used in each program. However, a common challenge in costing public health interventions in low- and middle-income countries that are implemented by government ministries is that institutional accounting databases are not readily available and shared [[17], [18], [19]]. In these instances, we applied the Strengthening Economic Evaluation for Multisectoral Strategies Nutrition Common Approach in collecting cost data, developed by the University of Washington, which outlines that, where expenditure data are not available, a microcosting approach is more appropriate [20]. This involves developing costing tools using a comprehensive list of costing “ingredients” (e.g., program inputs, involved personnel at all levels) and collecting unit cost data on these inputs through interviews and discussions with local accountants [21]. Quantities of each input are then determined through interview or observation, and in this way, total program cost calculations are developed.

Cost data were collected through available accounting records, monthly or key informant interviews, and reviewed by research staff for relevance. In Somalia and South Sudan, cost data were obtained through accounting records, whereas in Mali, a combined approach of accounting records and interviews was used. The program in Mali was implemented by the MoH; therefore, the study team did not have access to the MoH’s accounting systems. As a result, costs in Mali were collected via a microcosting ingredients approach, with data collected from site staff interviews [21]. Cost data were gathered from a purposively selected sample of 4 OTP/TSFP sites and both SCs in the study area, then applied to 4 additional OTP/TSFP sites matched on size and location(urban/peri-urban/rural).

Time allocation interviews were conducted to ascertain how staff time was allocated to CMAM programs. Time allocation data were collected for all staff involved in program implementation or management (see Section 1 of the Supplementary Materials for more details, including interview guides). Interviews with research staff were conducted to estimate the percentage of staff time and vehicle costs that were attributable to program implementation by influencing the quality and implementation of each CMAM program. A total of 37 interviews were conducted in South Sudan, 29 in Somalia, and 52 in Mali.

Data were collected from January 2021 to July 2022.

### Cost data compilation

Financial costs for the 3 CMAM programs were collected from institutional accounting systems. Grant accounting documents were reviewed for all 3 countries to identify expenditures relevant to CMAM implementation and support. Expenditures were categorized into the following groups (not all of which applied to each program): NGO Personnel, MoH Personnel, NGO Operating Costs, and MoH Operating Costs (Supplemental Materials, Supplemental Table 2). Cost centers for OTP/TSFP facilities included: facility running costs (rent and utilities), capital costs, supplies, and select staff: doctor, nurse, nutrition officer, community health workers (CHW), volunteers, logistics officer, security, cleaners. Volunteers received stipends as compensation for their time, eliminating the need for separate time-costing estimates. SC cost centers were the same and included the clinical supplies used in these facilities.

All costs were adjusted to USD in the institutional accounting systems using monthly exchange rates. Final cost estimates were adjusted for inflation using the Consumer Price Index [22] and are presented in 2022 USD. Costs of capital items were amortized using standard tables (3 y for computers and 5 y for all other equipment) and discounted at a rate of 3%.

Staff time allocation data were used to allocate institutional costs to the CMAM program. Monthly costs for each input line item were first adjusted for the percent of time allocated to the CMAM program, and then the percent of that time allocated to the study sites, across all the program sites (SC, OTP, and TSFP).

Although no children enrolled in the study were admitted to an SC, 10% of SC costs were allocated to the OTP programs and 5% to the TSFP programs, based on anticipated SC usage in each program, as SC services are essential for the successful implementation of CMAM, even if not utilized.

Costs for food products used to treat children were estimated using a bottom-up approach, calculated by multiplying the unit cost for a sachet of RUTF/RUSF by the number of sachets provided per child per visit throughout their treatment.

Costs were analyzed by group: personnel/other, program/support, and variable/fixed. “Support” costs referred to expenditures not directly related to specific program activities but instead focused on general program support. Variable costs were primarily related to supplies and food products, which vary with the number of admissions. Costs were also categorized by input types: staffing, security, program inputs, capital, overhead, and transport. For the purposes of this analysis, overhead refers to an expense related to operating a business that cannot be allocated to a particular activity, including office running costs, supplies, and communication; it does not refer to a general proportion of costs charged by an institution to donor agencies.

### Analytical approach

This study used cost-efficiency methods to calculate unit costs for different outpatient-based treatment episodes in CMAM service components. Episode types included SAM treatment to initial recovery, relapse to SAM treatment, and relapse to MAM treatment. Relapse to SAM treatment was defined as a child who recovered from SAM but subsequently relapsed to SAM within 6 mo postdischarge, requiring SAM treatment. Relapse to MAM treatment was defined as a child who recovered from SAM but later relapsed to MAM within 6 mo postdischarge, requiring treatment for MAM.

Monthly program costs were compiled and analyzed for costs attributable to cases occurring throughout each program. The mean cost per treatment episode was estimated for each country in a series of steps (Supplemental Materials, Supplemental Figure 2). First, mean monthly costs per CMAM service type (OTP, TSFP, or SC) were calculated and adjusted by the percentage of total caseload enrolled to get mean monthly costs per service component, including initial SAM treatment, relapse to SAM treatment, and relapse to MAM treatment. Second, the mean monthly cost per service component was adjusted by the number of children per service component to get an mean cost per child per service. Lastly, the mean monthly cost per child per service was adjusted by the mean length of stay per service component to determine the mean cost per treatment episode. To account for children with incomplete retreatment data, retreatment episode costs were based on the mean length of stay for completed treatments for relapse to MAM or SAM.

## Results

Among the 3 countries, Somalia’s CMAM program experienced the highest mean monthly caseload per facility at 273 [95% confidence interval (CI): 191, 356] and 774 (95% CI: 601, 934) for OTP and TSFP, followed by South Sudan with 56 (95% CI: 48, 63) and 228 (95% CI: 209, 245) for OTP and TSFP, respectively. Mali had the lowest per-facility caseloads, averaging 30 (95% CI: 27, 35) children in OTP and TSFP services combined (Table 1). A total of 1815 children discharged from CMAM after recovering from uncomplicated SAM were followed for 6 mo postdischarge: 403 in Mali, 800 in Somalia, and 612 in South Sudan. After initial SAM recovery, 115 children (29%) relapsed to AM in Mali, 32 (4%) in Somalia, and 278 (45%) in South Sudan. Among those who relapsed, 28 children (7%) in Mali, 16 (2%) in Somalia, and 44 (7%) in South Sudan relapsed to SAM (Table 1). Of the relapsed cases, 101 (88%) received retreatment in Mali, 22 (69%) in Somalia, and 193 (69%) in South Sudan (Table 1). A smaller proportion of children who relapsed to MAM received retreatment compared with those who relapsed to SAM (Table 1).

**TABLE 1:** CMAM caseloads, relapse rates, and length of stay in Mali, Somalia, and South Sudan

	Mali	Somalia	South Sudan
No. of participating facilities	9	1	6
Mean total monthly caseload per facility			
OTP treatment	14	273	56
TSFP treatment	16	774	228
Post initial SAM treatment outcomes	*N* = 403	*N* = 800	*N* = 612
Relapsed to SAM	28 (7%)	16 (2%)	44 (7%)
Received retreatment	26 (93%)	15 (94%)	42 (95%)
Mean # of retreatment episodes per child	0.0769	0.0188	0.0686
Relapsed to MAM	87 (22%)	16 (2%)	234 (38%)
Received retreatment	75 (86%)	7 (44%)	151 (65%)
Mean # of retreatment episodes per child	0.241	0.0175	0.225
Mean length of stay			
Treatment for initial SAM recovery (days)	*N* = 403	*N* = 800	*N* = 612
OTP	50	42	36
TSFP	—	37	68
Relapse to SAM treatment (days)	*N* = 26	*N* = 15	*N* = 42
OTP	68	24	51
TSFP	—	31	59
Relapse to MAM treatment (days)	*N* = 75	*N* = 7	*N* = 151
TSFP	45	37	55

Abbreviations: CMAM, community-based management of acute malnutrition; MAM, moderate acute malnutrition; OTP, outpatient therapeutic program; SAM, severe acute malnutrition; TSFP, targeted supplementary feeding programs.

The mean duration of initial SAM treatment to recovery was 50 d in Mali, 79 d in Somalia, and 103 d in South Sudan (Table 1). Among children requiring retreatment for SAM, the mean length of stay increased by an mean of 18 d in Mali and 7 d in South Sudan. Conversely, children in Somalia experienced a shorter retreatment duration – nearly 1 mo less than their initial treatment. For children who relapsed to MAM, mean retreatment durations ranged from 37 to 55 d, with the shortest mean stay observed in Somalia.

Total costs and costs per category for 19 mo of implementation for the 3 CMAM programs are presented in Table 2. Total program costs were similar in Mali and Somalia, ranging from $498,366 to $556,396, with costs in South Sudan being more than twice as high at $1,298,431. The percentage of total resources dedicated to support costs was similar in Mali and South Sudan (24%–26%) and slightly lower in Somalia (17%). Although this suggests a potentially more efficient program, due to the centralized nature of the CMAM program’s implementation in Somalia, it is not possible to declare this with certainty for several reasons. Delivering programs in large urban centers is typically more costly than in smaller centers in rural areas. Furthermore, opportunity costs for participation are usually lower in urban areas with higher population density, which may contribute to successful retention in the program. The percentage of variable costs was relatively high in Somalia (55% of total), where implementation occurred at 1 facility in an urban location and experienced the highest caseloads. Comparatively, the proportion of variable costs in Mali and South Sudan was lower (24% and 33%, respectively), given the multiple and dispersed facilities across geographically large rural areas. The percentage of total costs represented by products (e.g., RUTF/RUSF) follows the same trend as the percent of variable costs, as product costs constitute the majority of variable costs in CMAM programs. The proportion of costs by program input type is fairly consistent across countries, with program inputs and staffing costs comprising between 70% and 79% in each location.

**TABLE 2:** Total costs and cost categories for CMAM program implementation from January 2021 through July 2022 in Mali, Somalia, and South Sudan

	Mali	Somalia	South Sudan
Total cost (program and product)	$498,366	$556,396	$1,298,431
Product (RUTF/RUSF) cost	$66,177	$231,945	$404,940
Product % of total cost	13	42	31
% Support^1^	24	17	26
% Variable^1^	24	55	33
Input type^2^:			
Staffing	45%	28%	39%
Security	1%	0%^3^	15%
Program inputs	34%	42%	38%
Capital	0%	0%	1%
Overheads	19%	20%	2%
Transport	2%	10%	6%

Abbreviations: CMAM, community-based management of acute malnutrition; RUSF, ready-to-use supplementary foods; RUTF, ready-to-use therapeutic foods.

1 Percentage of support and variable costs are estimated separately and will not sum to 100%.

2 Input %s may not sum to 100% due to rounding.

3 Security costs in Somalia were 0.3% of total costs, and the decimals were rounded for presentation in this table.

The calculation of monthly costs and their adjustments for analysis are presented in the Supplemental Materials (Supplemental Table 3). These calculations were used to estimate the mean cost per treatment episode. Table 3 presents the mean cost per treatment episode for different CMAM service components (e.g., SAM treatment in OTP and TSFP or OTP alone; MAM treatment in TSFP), along with the total cost per child failing to sustain recovery and the mean cost of relapse treatment across the population of children enrolled in the parent study in each country.

**TABLE 3:** Mean costs^1^ of initial treatment and various relapse treatments in CMAM programs in Mali, Somalia, and South Sudan

	Mali	Somalia	South Sudan
Mean cost of treatment episode			
(A) Cost per child recovered in initial SAM treatment^2^	$191	$92	$178
(B) Cost per child treated for relapse to SAM	$215	$64	$215
(C) Cost per child treated for relapse to MAM	$118	$27	$50
Total cost per child failing to sustain recovery			
(D) Cost per child recovered and subsequent treatment for relapse to MAM (A + C)	$309	$119	$228
(E) Cost per child recovered and subsequent treatment for relapse to SAM (A + B)	$406	$156	$393
Ratio of cost per failure to sustain recovery to sustained recovery			
(F) Relapse to MAM: sustained recovery (D/A)	1.6	1.3	1.3
(G) Relapse to SAM: sustained recovery (E/A)	2.1	1.7	2.2
Mean cost of relapse per admitted child (including those not requiring retreatment themselves)			
(H) Total cost of relapse per child (including cost of initial treatment) (adjusted for mean # episodes/service/country)	$236	$94	$203
(I) Additional cost of relapse per child (excluding cost of initial treatment) (adjusted for mean # episodes/service/country)	$45	$2	$26

1 2022 USD.

2 Equivalent to cost per child recovered, because only children in initial SAM treatment who recovered were included in the relapse analysis.

Treatment costs for relapse to SAM episodes (row B) were higher on mean compared with initial SAM treatment. Rows D and E show the total costs per child who failed to sustain recovery, disaggregated by relapse to MAM (row D) and SAM (row E) treatment. These show that children who fail to sustain initial recovery and relapse to SAM require a resource expenditure of between $156 and $406. On mean, this is nearly double the cost of treating a child who recovers from SAM and maintains recovery without relapse (row G). The estimated total costs of children who relapsed to MAM, inclusive of their initial treatment, ranged from $119 to $309. Not all children in each country experienced relapse. When adjusting for the mean number of episodes per child, the total cost of relapse, including initial treatment (row H), was between $94 and $236 per child. This suggests that by preventing relapse, an additional $2–$45 per child would be made available per child (row I). Table 1 presents the mean number of episodes per child per country that were used in these calculations.

## Discussion

This study assessed the economic implications of relapse after initial SAM recovery in CMAM programs in Mali, Somalia, and South Sudan. The findings are intended to raise awareness within the global nutrition community about the costs of treating children who relapse after recovery from SAM and to strengthen efforts toward relapse prevention.

Our estimated cost per child recovered from initial SAM treatment ($92–$191) falls within the range reported by similar studies ($165–$363) that accounted for comprehensive costs, including shared costs, management, and infrastructure (Supplemental Materials, Supplemental Table 4). Although consistent with the existing literature, overall cost structures differed by country based on the distinct setting of each study location, providing an evidence base relevant to a variety of implementation settings.

Somalia had the lowest overall and unit costs. This was mostly driven by higher coverage, enabled by the density of the population in a significantly smaller geographic setting (i.e., urban IDP camp), lower relapse rates, efficient operations (the concentration of services in a single facility operating 6 d/wk), and the highest caseload among all study countries. The setting in Somalia, which enabled these economies of scale, is unique. Most contexts with a high AM burden require CMAM models similar to rural Mali and South Sudan. The model in Somalia demonstrates that CMAM costs are lower when services are centrally located and fixed costs are minimal relative to caseload. Conversely, the more rural settings of South Sudan and Mali require multiple facilities across a large geographic area, with lower caseload per facility leading to a higher share of fixed costs. This suggests that higher site-specific caseload and a lower proportion of fixed costs play a crucial role in achieving lower unit costs of treatment. Similarly, other public health studies have shown economies of scale gained with lower unit costs when expanding coverage and increasing caseloads [[23], [24], [25]]. However, the literature also notes that economies of scale have a limit and that costs do not scale linearly, especially when ensuring universal access for remote communities. For example, the World Bank estimates that although a program may reach 80% population coverage at a constant marginal unit cost, extending coverage by an additional 10% could double that cost [26].

Specifically in the CMAM literature, expanding coverage by delivering SAM treatment through CHW has been identified as an important factor in treating more children, lowering unit costs, and potentially serving as an effective platform for addressing relapse [27]. However, increasing the number of children reached through smaller points of treatment, rather than 1 high-caseload facility, can result in what looks like inefficiencies in unit costs. Two studies conducted by Cichon et al. [28,29] found lower unit costs in a facility-based program in Niger compared with a CHW-delivered program in Mali. This indicates that having a multitude of smaller satellite sites can improve coverage, but also results in what might appear to be inefficiencies in costing, at least in the short term with fewer children admitted to a larger number of providers. Different program staffing profiles did not appear to drive costs in the present study [45% personnel cost share in Mali (MoH-run), 39% in South Sudan (NGO-run); Table 2].

Regarding postdischarge outcomes, this analysis found that retreatment of relapse to SAM was 13%–21% more expensive in Mali and South Sudan compared with treatment for the initial SAM episode. Furthermore, programs spent more on mean treating relapse to MAM than relapse to SAM, because of the higher rate of relapse to MAM compared with relapse to SAM. This may be influenced by both the study’s follow-up frequency and the typical progression of AM cases: presenting first as MAM before deteriorating to SAM. These findings suggest that by preventing relapse, an mean of $2/child could be saved in Somalia, $26 in South Sudan, and $45 in Mali, compared with the additional $30–$200 spent on retreatment per child who relapses. Strengthening support for sustaining recovery could free up substantial resources, which could be redirected toward relapse prevention or expanding treatment access for newly-affected children. In Mali and South Sudan, these savings would be adequate to contribute to preventive programming that has been proven to be effective and efficient, potentially including complementary feeding promotion ($19–62) [30] or provision of small quantity lipid nutrient supplements for 12 mo ($52 in Uganda) [31], among others.

Although we did not calculate the proportion of program costs allocated to relapse due to inadequate information to do so, we note a few considerations for future studies. A key factor in interpreting the cost of relapse treatment is understanding how program performance influences the distribution of costs. When recovery rates are low, fewer children are classified as “recovered”; therefore, fewer children are eligible to experience a postrecovery relapse, resulting in a smaller proportion of total program costs being allocated to the retreatment of recently recovered children. Such children may be discharged as “non-responders” or “defaulters” but are later readmitted as a new admission, despite it being a type of retreatment. Conversely, higher recovery rates increase the pool of children at risk of relapse, potentially raising the share of costs associated with treating relapsed cases. However, the literature suggests that CMAM programs with lower recovery rates are likely to see higher relapse rates [32,33]. Such combination of poor indicators signifies that the CMAM program is likely implemented in a particularly adverse context, such as high food insecurity, low access to other healthcare services, minimal other humanitarian assistance services, conflict, etc. This pattern was evident in this study, with Somalia’s CMAM program reporting the highest recovery rates, the lowest relapse rates, the shortest lengths of stay, and the lowest cost of relapse per child.

In addition, in typical program settings, not all children who experience a biological relapse will seek retreatment. In this study, our researchers implemented very close postdischarge monitoring with immediate referral for retreatment. In real-world settings, routine postdischarge monitoring and referrals are limited or nonexistent. Therefore, the proportion of funds required to address relapse in a program may be underestimated.

The indicators and thresholds used to define AM—and ultimately determining admission and discharge criteria—also underpin the cost of relapse treatment. In this study, the 3 programs followed distinct CMAM protocols, directly influencing caseloads, recovery criteria, and the classification of relapse and retreatment eligibility. Modifying admission and discharge criteria can significantly impact relapse rates, as children may or may not meet revised readmission thresholds. In the parent study, applying the broader 2023 WHO definition of AM resulted in relapse rates ≤34 percentage points higher than some of the less conservative country-specific program criteria [34]. Going forward, if more countries adopt the 2023 WHO admission and discharge criteria, more children are likely to qualify for retreatment, possibly increasing the share of costs allocated to relapse retreatment. Conversely, limiting the criteria of CMAM programs to MUAC only (a more restrictive definition of AM) would reduce relapse rates, potentially decreasing the associated costs of retreatment. Moreover, changes in AM definitions can influence length of stay and recovery outcomes, as stricter exit criteria may prolong treatment influencing both initial and relapse-related costs.

Due to the extensive assumptions required for key input variables, the proportion of total program costs attributable to relapse was not calculated in this study. As discussed, even slight variations in program implementation or performance can substantially influence relapse-related costs and their contribution to overall program expenditures. Future studies are needed to develop a sophisticated modeling approach capable of generating scenario-based simulations that incorporate multiple uncertainties and dynamic program parameters, including detailed data not only on treatment and postdischarge outcomes of children who recovered from initial SAM, but also for those who did not recover and those who received MAM treatment only.

### Limitations

This study had a few limitations. First is the short follow-up, of only 6 mo, after discharge from initial SAM treatment. This limitation likely translates to an underestimation of costs spent on retreatment for SAM relapse compared with what is captured here. Furthermore, for children who relapsed closer to the latter end of those 6 mo or whose treatment for relapse comprised lengths of stay that extended beyond that 6-mo postdischarge time point, we were unable to capture the full extent of their relapse costs. This applied to only 10 children in South Sudan, representing 5.18% of children who relapsed and received retreatment there. All children in Somalia and Mali were followed for the full duration of their retreatment. For the South Sudan cases, we assumed the mean length of stay for all children who relapsed.

Second, given the high frequency of post-SAM monitoring visits, we likely observed more relapse to MAM (as opposed to SAM). Without such close monitoring, children may have continued to regress from MAM to SAM, leading to higher rates of relapse to SAM. Other literature suggests that where MAM treatment through TSFP is unavailable, relapse to SAM rates tend to be higher [[35], [36]]. Therefore, our estimates of costs associated with treating relapse to SAM are likely underestimated compared with what occurs in nonstudy settings.

A third limitation results from differences in program implementing partners. Cost data in Mali was collected through interviews, whereas data from Somalia and South Sudan were collected via accounting records. Therefore, we were likely unable to capture as broad a set of costs in Mali as the other 2 countries.

Fourth, we present RUTF/RUSF product cost data using a bottom-up cost calculation; therefore, our product cost estimates do not account for product wasted in spoilage and leakage and instead reflect the normative amount to be provided per child. Finally, because our analysis focused on facility-level treatment-specific costs, we excluded some of the preventive costs that are typically part of CMAM programs, primarily community sensitization costs. This could result, to some extent, in under-reporting of costs attributable to CMAM programming.

In conclusion, this analysis is the first to estimate the cost of retreating children who relapse to AM within 6 mo of SAM recovery. We found that the cost per child who failed to sustain recovery and required treatment for relapse to SAM was, on mean, nearly double that of a child who sustained recovery. These findings highlight a significant opportunity for cost savings: preventing relapse not only improves child health outcomes but also frees up valuable resources that could be reinvested in preventative interventions or in addressing any longer-term sequelae from having experienced AM.

In the current context of shrinking foreign aid budgets and increasing pressure on humanitarian funding, these results offer a compelling economic rationale for strengthening the efficiency of global nutrition programs. Investing in strategies that enhance the effectiveness of CMAM, such as more robust postdischarge support, targeted prevention efforts, and early identification of at-risk children, could reduce relapse rates and maximize the impact of existing resources.

## Author contributions

The authors’ responsibilities were as follows – CP, HS: conceptualized the study design and oversaw the study; SK: compiled cost data and made substantial contributions to the acquisition of the data; SMC, FAM, NL: made substantial contributions to the acquisition of the data; CP, SK: analyzed the data and prepared the manuscript; HS: substantively revised the manuscript; CP, SK, HS: interpreted the data and were responsible for final content; and all authors: read and approved the final manuscript.

## Funding

The United States Agency for International Development provided financial support for this article through the Bureau of Humanitarian Assistance. It was prepared under the terms of contract 720FDA19GR00278 awarded to Action Against Hunger United States. The contents are the responsibility of the authors and do not necessarily reflect the official position of USAID or the United States Centers for Disease Control and Prevention. The funding agencies had no role in the study design, data collection, analysis and interpretation of data, or in writing the manuscript.

## Ethics committee approval

Ethical approval was provided by Solutions Institutional Review Board (#20200310), the London School of Hygiene and Tropical Medicine’s Research Ethics Committee (#18059), the Ministry of Health and Human Services of Somalia (MOH&HS/DGO/0429/03/202), the Université Des Sciences, Des Techniques Et Des Technologies De Bamako (2020/202/CE/FMOS/FAPH)) in Mali, and the Ministry of Health of South Sudan (MOH/ERB6/2020).

## Conflict of interest

CP reports financial support was provided by Action Against Hunger. The other authors declare that they have no known competing financial interests or personal relationships that could have appeared to influence the work reported in this article.
