# Supplementary material for: The Economic Implications of Relapse Among Children Recovered from Severe Acute Malnutrition: Results from a Multicountry Prospective Study in Mali, Somalia, and South Sudan
Source: Curr Dev Nutr. 2025 Dec 10;10(1):107616. doi: 10.1016/j.cdnut.2025.107616 (PMC12808978; doi:10.1016/j.cdnut.2025.107616)
Supplement: Multimedia component 1 [file mmc1.docx]

SUPPLEMENTARY MATERIAL

For manuscript titled

The economic implications of relapse among children recovered from severe acute malnutrition: Results from a multi-country prospective study in Mali, Somalia, and South Sudan

By Chloe Puett, et al.

Contents

[Section 1: Time allocation methods 1](#_Toc210219282)

[NGO staff 1](#_Toc210219283)

[MoH Staff 3](#_Toc210219284)

[Section 2: Key assumptions used 5](#_Toc210219285)

[Section 3: Indicators and equations used in analyses 6](#_Toc210219286)

[Supplementary Tables 10](#_Toc210219287)

# Section 1: Time allocation methods

Time allocation of all staff involved in program implementation and management was measured using self-report during key informant interviews with semi-structured questionnaires performed by trained Research staff. This method was selected due to constraints on resources and logistics making continuous observation or time-motion methods infeasible (Huebschmann et al.). One research staff person per country was trained on time allocation interview methods in December 2021. The training focused on recording data and interview techniques to ensure accuracy of responses. Different tools were used for NGO and MOH staff. An interview guide is included below.

**Time allocation interview guide**

## NGO staff

This interview is a part of the cost analysis that is being conducted for the Relapse study by the Research department at Action Against Hunger (“NGO”) across 3 country offices: Mali, Somalia and South Sudan. The purpose of the costing analysis is to estimate the average cost of treatment for acutely malnourished children, by both ACF and MoH in the 3 countries, in order to estimate the additional cost of repeating treatment for children who have relapsed. The results of the analysis will show how much more it costs to treat a relapsed case, beyond the cost of initial treatment. This information will be used to advocate for efforts to reduce relapse by improving the adherence, coverage, uptake and eventual effectiveness of initial treatment for acute malnutrition.

I am going to ask you some questions about the time that you spend on services for acute malnutrition that you are involved in as part of your work. This information is not being collected to evaluate how much you work or the quality of your work, but instead to understand how much staff time is being spent on different activities so we can apply an accurate cost of your time for the costing study.

**For implementation & management staff**

On average, how many hours per week do you work?

(Walk through usual hours per day of the week and add up)

How many days (or hours) of this time is spent on CMAM activities, including OTP and TSFP?

(Take their answer, get the corresponding % time and tell it back to them. For example, if they say they work 8 hours (1 day) per week on CMAM, confirm with them. “So, you think you spend about 20% of your time or 1 day per week on CMAM?” This may help them to refine their answer).

So, you think you spend about __% or __ day(s) per week on CMAM? (Record their final % to CMAM and non-CMAM.)

Of the time you spend specifically on CMAM, what proportion of time is spent on OTP, TSFP, and PLW services? Record their final % time to CMAM sub-activities.

For Research Staff: What proportion of your time do you spend on activities that either support the Operations team or could influence program quality, instead of just observing it? (These will be the operations support activities.)

**For support staff**

(This section is for staff who are not involved in program activities, such as Finance, HR, Logs, CD, etc.)

How many programs are currently being run at this office? (Listen to their list of programs, make a list of them and note the total number)

Do all of these programs take the same amount of time for you to support? (If “Yes”, divide 1 by the total # of programs and this is the % time per program. If “No”, revise the estimates for the various programs up or down until they are satisfied with the percentages.)

So, you think you spend about __% or __ day(s) per week on CMAM? (Record their final % to CMAM and non-CMAM in **columns B&C**. This amount of time should then be evenly split among the different CMAM sub-activities being implemented at your country office.)

## MoH Staff

This interview is a part of the cost analysis that is being conducted for the Relapse study by Action Against Hunger. The purpose of the costing analysis is to estimate the average cost of treatment for acutely malnourished children, by both ACF and MoH, in order to estimate the additional cost of repeating treatment for children who have relapsed. The results of the analysis will show how much more it costs to treat a relapsed case, beyond the cost of initial treatment. This information will be used to advocate for efforts to reduce relapse by improving the adherence, coverage, uptake and eventual effectiveness of initial treatment for acute malnutrition.

**Case management tab**

I am going to ask you some questions about your time allocation to services for acute malnutrition that you are involved in as part of your work. This information is not being collected to evaluate how much you work or the quality of your work, but instead to understand how much staff time is being spent on different activities so we can apply an accurate cost of your time for the costing study.

Which services are you involved in?

- OTP
- TSFP
- SC

OTP:

How many children are admitted to OTP per month on average? (Ask them also to try to give an average if possible.)

How many times per month are OTP sessions offered at this facility?

How much time do you spent with each child admitted to OTP? (Ask them also to try to give an average if possible.) OR if recording length of total OTP session instead of time with each child, ask “How much time does each OTP session last?”

Do you spend any additional time per month on OTP, beyond time with patients, such as for reporting or other related administrative tasks?

TSFP:

How many children are admitted to TSFP per month on average? (Ask them also to try to give an average if possible.)

How many times per month are TSFP sessions offered at this facility?

How much time do you spent with each child admitted to TSFP? (Ask them also to try to give an average if possible.) OR if recording length of total OTP session instead of time with each child, ask “How much time does each TSFP session last?”

Do you spend any additional time per month on TSFP, beyond time with patients, such as for reporting or other related administrative tasks?

SC:

How many children are admitted to SC per month on average? (Record any details on range, etc.)

What proportion of the admissions to SC are under 6 months old?

What is the average number of days spent at the facility per child admitted to SC?

How much time do you spend admitting each case to SC?

How much time, on average, do you spend managing each case at SC during the day (24-hour period)? (i.e., # of visits to their bed and time spent per visit. Ask them also to try to give an average if possible.)

How much time do you spend discharging each case from SC?

Do you spend any additional time per month on SC, beyond time with patients, such as for reporting or other related administrative tasks?

All staff:

How many hours do you work per week, on all activities at the health facility, on average? We are not trying to judge how much you work, but to get an average number so we can estimate a percent of your time allocation for CMAM.

How many days do you work per month, on average?

**Other activity time tab**:

What are some of the other activities related to acute malnutrition that you work on, on a monthly or weekly basis? (Get a list of activities per staff. For each activity, ask the following questions, walking them through their time allocation, getting the frequency of the activity and how much time it takes them per session.)

How often does this activity take place? OR, “How many days per week/month do you perform this activity?”

How much time does this activity take, each time you perform it?

**MoH salary list tab:**

Discuss with the MoH accountant about the total annual salary (including annualized bonuses and benefits) for each of the staff involved in implementing or supporting AM-related activities at the health facility.

**Medical inputs per child tab:**

Can you tell me on average, what medicines and medical supplies are given to each child admitted to OTP, TSFP and SC during their course of treatment? (Be sure to get the number of units used per child for each item.)

Can you give me a unit cost for each of these items?

(If the facility has a SC center) Can you tell me the approximate cost per bed per day as estimated by the health facility, and the cost of food per day given to each child’s caretaker?

**Community interviews tab:**

(To discuss with any volunteers, CHWs or other relevant community members) We are conducting a costing analysis for the Relapse study by Action Against Hunger. The purpose of the costing analysis is to estimate the average cost of treatment for acutely malnourished children, by both ACF and MoH, in order to estimate the additional cost of repeating treatment for children who have relapsed. The results of the analysis will show how much more it costs to treat a case that receives multiple rounds of treatment due to relapsing, beyond the cost of initial treatment. This information will be used to advocate for efforts to reduce relapse by improving the adherence, coverage, uptake and eventual effectiveness of initial treatment for acute malnutrition.

As part of this, we would like to include an estimate of the value of time contributed by community-level staff. We are looking to find an estimate of the value of these staff people’s time and have a couple of questions.

Would people who work as community health workers or community nutrition volunteers also potentially work in agricultural daily labor?

In your community, what is the daily agricultural wage offered to laborers?

Are there any other daily wage estimates that could be relevant for this kind of staff?

References for this section:

Huebschmann AG, Trinkley KE, Gritz M, Glasgow RE. Pragmatic considerations and approaches for measuring staff time as an implementation cost in health systems and clinics: key issues and applied examples. Implementation science communications. 2022 Apr 15;3(1):44.

# Section 2: Key assumptions used

- **Program scope**: Only costs linked to CMAM service delivery are included; preventive/community sensitization excluded.
- **Currency/inflation handling**: All costs were adjusted to USD in the institutional accounting systems using monthly exchange rates. Final cost estimates were adjusted for inflation using the Consumer Price Index from the United States Bureau of Labor Statistics and are presented in 2022 USD.
- **Capital costs**: Lifespan assumptions (3 years for computers, 5 years for other equipment) and 3% discount rate.
- **Staff time allocation**: Derived from self-reported interviews; proportion of staff time attributable to CMAM versus other duties.
- **Relapse treatment length**: Average length of stay imputed for children with incomplete follow-up.
- **Costing of products (RUTF/RUSF)**: Based on normative provision, not accounting for spoilage or leakage.
- **Caseload denominator**: Relapse calculations restricted to children followed in the parent study; relapse treatment beyond 6 months not captured.
- **Definitions of admission/recovery/relapse**: Based on country-specific CMAM protocols, not harmonized across settings.
- **SC cost apportioning**: While no children enrolled in this study used SC services, a proportion of the costs of SC were included in each country since this is a necessary resource involved in CMAM programming. These were included at 10% of OTP costs and 5% of TSFP costs. These proportions were used as this is the proportion of caseloads for each service type that are anticipated potentially to need SC services, as a rule of thumb based on expert opinion.

# Section 3: Indicators and equations used in analyses

This section has been replicated from the study protocol, with the following reference: Puett, C., King, S., & Stobaugh, H. (2022). A multi-country, prospective cohort study to evaluate the economic implications of relapse among children recovered from severe acute malnutrition: a study protocol. *BMC Nutrition*, 8(1), 139.

**Data Analysis**

Indicators to be used in the analysis are described in the following sections and an indicator matrix is outlined in **Table 3**.

*Monthly program costs per service component*

Total program cost estimates will be compiled on a monthly basis during the study timeframe. Total costs will be apportioned to each CMAM service component (e.g., OTP, SFP, SC) according to allocations established through interviews and accountancy data to determine monthly costs associated with each service component provided.

*Monthly cost per child per service component*

Using monthly CMAM program data that details the total number of children receiving care in each of the CMAM service components, the total costs per service per month will be divided by the number of children in each service for each month. This will estimate the average monthly cost of treatment per child in each service. For example, the calculation for OTP in Month “A” will be as follows:

Total Month “A” OTP Service Costs

Total No. Children in OTP in Month “A”

**=**

Month “A” total OTP
costs per child

Each of the monthly costs per child per service type will then be added across the life of the study and divided by the total number of months of program implementation during the study to determine an average monthly cost per child per service type.

*Cost per initial SAM recovery*

Using programmatic data, the average length of stay (LoS) in each of the different CMAM service components will be calculated in fractions of months for study children who are admitted for initial SAM treatment and recover. Since many children transfer across CMAM service components, the cost of each component will be added together to encompass the total CMAM services provided for the entire LoS for that episode’s treatment. For example, we will calculate that, on average, a child who recovers from an initial SAM episode spends “X” months in OTP plus “Y” months in SFP. The calculation for the cost per initial SAM recovery will be as follows:

$${c_{t}=\overline{c}}_{OTP}\overline{L}_{OTP}+\overline{c}_{SFP}\overline{L}_{SFP}$$

Where C_t_ is the cost per initial SAM recovery, C_OTP_ and C_SFP_ are the average monthly costs of OTP and TSFP per child, respectively, and L_OTP_ and L_SFP_ are the average length of stay (in fractions of months) in OTP and SFP of children who recover from initial SAM treatment.

*Cost per SAM child treated and SAM child recovered*

To produce indicators comparable to other CMAM costing studies, we will also calculate unit costs for SAM treatment outcomes. These indicators will also be used as measures of program performance to compare across the three study countries. This includes the cost of children initially treated for SAM regardless of discharge outcome and the cost of those who are discharged as recovered. These will be analyzed in two ways: First, an average cost per child treated will be calculated as total number of children treated in initial SAM treatment divided by the total cost of initial SAM treatment across the duration of program implementation.

Total Number of Children Receiving Initial SAM Treatment

Total Cost of Initial SAM Treatment Across Duration of Program

Average cost per SAM child treated

**=**

Second, the cost of only the recovered children (who were eligible for inclusion in the larger SAM Relapse Study) will be calculated as the total number of children discharged as recovered divided by the total cost of initial SAM treatment across the duration of the program implementation.

Total Number of Children Discharged as Recovered in Initial SAM Treatment

Total Cost of Initial SAM Treatment Across Duration of Program

Average cost per child recovered

**=**

*Cost of child who fails to sustain recovery*

The total cost incurred by a child who fails to sustain recovery for six-months following initial SAM treatment will build upon formulas presented above and additional data from the parent SAM Relapse Study. For those who relapsed, the average monthly cost per retreatment per service type will be calculated. This will then be multiplied by the average LoS (in fractions of months) per each of the service types (e.g., OTP, SFP, and SC) throughout the entire six-month post-discharge follow-up period. Those who relapse may spend time in the SC, which will also be included in the calculation if applicable. Conversely, if a child relapses and only spends time in the SFP, then only this time will be applied. This calculation will account for all relapse episodes if and where multiple relapses occurred. The computation is as follows:

$$\overline{C}_{r}{=\bar{c}}_{OTP} \bar{L}_{OTP}+\bar{c}_{SFP} \overline{L}_{SFP}+\bar{c}_{SC}\bar{L}_{SC}$$

Where C_r_ is the average cost of retreatment for relapse during the six-month post-discharge period, C_OTP_, C_SFP_ and C_SC_ are the average monthly costs of OTP, SFP and SC per child, respectively, and L_OTP_, L_SFP_ and L_SC_ are the average length of stay (in fractions of months) in OTP, SFP, and SC of children who require retreatment.

It is important to note that the length of stay in treatment for children who relapse will likely be shorter in this study than in a non-study setting. Because study resources only allow for a six-month post-discharge follow-up period, many of the children who relapse will likely still be receiving treatment by the end of the study (or the end of the six-month post-discharge follow-up period). Thus, the average length of stay will not necessarily account for the full time a child is treated until discharge of a relapse episode.

The cost of retreatment for relapse will then be added to the cost per child recovered from initial SAM treatment, in order to encompass the cost of treating a child for SAM and the costs of retreating the same child for relapse(s) during the subsequent six months. The calculation is:

$$\overline{C}_{f}=\sum\overline{C}_{i} \overline{C}_{r}$$

Where C_f_ (the average cost per child who fails to sustain recovery) equals the sum of the average cost per child recovered during initial SAM treatment (C_i_) and the average cost of retreatment for relapse during the six-month post-discharge period (C_r_).

Study data will be explored for the possible identification of certain patterns of relapse pathways in each country which may allow for appropriate and relevant disaggregation of results.

*Cost efficiency analysis*

The unit cost estimates outlined above will enable calculation and comparison of the average cost of treating a child of SAM that relapses after initial CMAM treatment with the cost of a child with SAM that remains recovered for six months post-discharge. It will also allow for the comparison of an average cost of initial episode of SAM treatment with an average cost of relapse episode of SAM retreatment.

**Table 3. Indicator matrix (table numbering from original article)**

| **Indicator(s)** | **Definition/description** | **Data Collection Method** | **Time period** |
| --- | --- | --- | --- |
| Monthly cost per child per CMAM service component | Average monthly cost incurred per child in each service (SC, OTP, SFP). Estimated as: the total costs per service per month divided by the number of children who were enrolled in each service per month | Review of financial records,  off-budget costs collected via interviews,  time allocation interviews,  study data, and CMAM program records | Monthly |
| Length of Stay (LoS) per child per CMAM service component | Average length of stay in each service, defined as the time from admission to discharge in each SC, OTP, and SFP separately. (When a child transfers from one component to another, this equates to a “discharge” and marks the end of his stay in that component of the CMAM program.) This will be estimated in months (and fractions of months) and calculated for initial SAM episode treatment, relapse to AM episode retreatment, and time in retreatment for any and all episodes of relapse to AM during the six months following initial SAM recovery. | study data; and CMAM program records | Entire study period |
| Average cost per child for initial SAM recovery | The overall cost to recover children enrolled in treatment for initial episode of SAM. Calculated using methods comparable to standard CMAM costing studies that do not account for relapse. Estimated as: the total costs of initial SAM treatment divided by the number of children recovered in initial SAM treatment. | Review of financial records,  off-budget costs collected via interviews,  time allocation interviews,  study data, and CMAM program records | Entire study period |
| Average cost of initial treatment for study eligible children | The cost per initial treatment episode (only for children recovered who were eligible for inclusion in the study) to be used in estimating total per-child treatment costs. This represents the average cost of treating a child with SAM who does not relapse. Estimated as: total cost of initial SAM treatment multiplied by the proportion of children recovered in initial treatment, divided by the number of children recovered in initial SAM treatment. | Review of financial records  Off-budget costs collected via interview  Time allocation interviews  # of children enrolled per service and LoS from study records | Entire study period |
| Average cost of retreatment for relapses during six-month post-discharge period | The average cost of retreatment for any and all relapse(s) to AM episodes within six months post-discharge from initial recovery. Estimated as: monthly cost per child per service multiplied by the average LoS in each service (in months) and for relapsed children. | Review of financial records,  off-budget costs collected via interviews,  time allocation interviews,  study data, and CMAM program records | Entire study period |
| Total average cost of relapse | The average total cost incurred for treatment of a child who relapses fails to sustain recovery for at least six months post initial recovery. Estimated as: cost of initial treatment + cost of retreatment for relapses during six-month post-discharge period. | Review of financial records,  off-budget costs collected via interviews,  time allocation interviews,  study data, and CMAM program records | Entire study period |

# Supplementary Tables and Figures

**Supplemental Table 1: Country Context and Treatment Protocols**

|  |  | **South Sudan** | **Somalia** | **Mali** |
| --- | --- | --- | --- | --- |
| **Context** | **Location** | Rural | Urban | Rural |
|  | **Population** | Majority permanent residents | Majority internally displaced population | Majority permanent residents |
|  | **No. of facilities** | 6 | 1 | 9 |
|  | **Operated by** | Action Against Hunger | Ministry of Health  & Action Against Hunger | Ministry of Health |
| **SAM Treatment in CMAM Program** | **SAM Admission Anthropometric Criteria^i^** | WHZ (<-3), MUAC (<115mm), and/or  bi-lateral pitting oedema | MUAC (<115mm) and/or  bi-lateral pitting oedema | WHZ (<-3), MUAC (<115mm), and/or  bi-lateral pitting oedema |
|  | **Anthropometric criteria used to monitor at intermediate follow-up visits^i^** | Either WHZ or MUAC according to which indicator was used for admission; if  both met admission criteria, then MUAC was used to track progress | MUAC | Both WHZ and MUAC |
|  | **SAM Discharge Anthropometric Criteria^i^** | Either WHZ (≥-2) or MUAC (≥125mm) (and no oedema) for two consecutive visits, determined by monitoring criteria | MUAC (≥125mm) (and no oedema) for two consecutive visits | Both WHZ (≥-1.5) and MUAC (≥125mm) (and no oedema) for two consecutive visits |
|  | **Transfer from OTP to SFP?** | Yes. Transferred from OTP to TSFP  when nutrition status changed from  SAM to MAM | Yes. Transferred from OTP to TSFP when nutrition status changed from SAM to MAM | No. Treated in OTP for duration of stay |
|  | **Follow-up visit frequency** | Weekly for OTP Fortnightly for TSFP | Weekly for OTP Fortnightly for TSFP | Weekly |
|  | **Products Used** | RUTF for OTP RUSF/CSB++ for TSFP^ii^ | RUTF for OTP RUSF for TSFP | RUTF for OTP |
|  | **Maximum Length of Stay** | Six months | Eight Months | Three months |
| **MAM Treatment in CMAM Program** | **MAM Admission Anthropometric Criteria** | WHZ (>-3 to <-2) and/or MUAC (>115mm to <125mm) | MUAC (>115mm to <125mm) | WHZ (>-3 to <-2) and/or MUAC (>115m to <125mm) |
|  | **Anthropometric criteria used to monitor at intermediate follow-up visits** | Either WHZ or MUAC according to which indicator was used for admission; if  both met admission criteria, then MUAC was used to track progress | MUAC | Both WHZ and MUAC |
|  | **MAM Discharge Anthropometric Criteria** | Either WHZ (≥-2) or MUAC (≥125mm) for two consecutive visits, determined by monitoring criteria | MUAC (≥125mm) for two consecutive visits | Both WHZ (≥-1.5) and MUAC (≥125mm) for two consecutive visits |
|  | **Follow-up visit frequency** | Fortnightly | Fortnightly | Fortnightly |
|  | **Products Used** | RUSF | RUSF | RUSF |
|  | **Maximum Length of Stay** | Three months | Four Months | Three months |

AM=acute malnutrition. CMAM=community-based management of acute malnutrition. CSB++=super cereal. MAM=moderate acute malnutrition. MUAC=mid-upper arm circumference. OTP=outpatient therapeutic programme. RUSF=ready-to-use supplementary food. RUTF=ready-to-use therapeutic food. SAM=severe acute malnutrition. TSFP=therapeutic supplementary feeding programme. WHZ=weight-for-height z-score.

^i^ Oedema was an admission criterion in all CMAM programs. Children must have had no oedema for two consecutive visits to be discharged.

^ii^ CSB++ was only used in TSFP when RUSF stockouts

**Supplemental Figure 1: Country Treatment Pathways**

**SOMALIA**

**MALI**

Recovered from SAM treatment and enrolled into study

**N = 403**

Relapsed to

SAM

**N = 28**

Relapsed to

MAM

**N = 87**

Received retreatment for

SAM

**N = 26**

Received retreatment for

MAM

**N = 75**

Recovered from SAM treatment and enrolled into study

**N = 800**

Relapsed to

SAM

**N = 16**

Relapsed to

MAM

**N = 16**

Received retreatment for

SAM

**N = 15**

Received retreatment for

MAM

**N = 7**

Recovered from SAM treatment and enrolled into study

**N = 612**

Relapsed to

SAM

**N = 44**

Relapsed to

MAM

**N = 234**

Received retreatment for

SAM

**N = 42**

Received retreatment for

MAM

**N = 151**

**SOUTH SUDAN**

**Supplemental Table 2: Cost categories by input and country**

| **Cost category** | **Mali** | **Somalia** | **SSD** |
| --- | --- | --- | --- |
| Personnel NGO |  |  |  |
| --Program Management | X | X | X |
| --Support Staff (national/field level) | X | X | X |
| --National Coordination | X | X | X |
| --Relapse study staff (time attributable to implementation-related quality measures) | X | X | X |
| --OTP/TSFP |  | X | X |
| --SC |  | X | X |
| Personnel MoH |  |  |  |
| --OTP/TSFP care providers | X |  |  |
| --SC care providers | X |  |  |
| --OTP/TSFP Support staff (Logistics officer, data clerk, security, cleaners) | X |  |  |
| --SC Support staff (security, cleaners) | X |  |  |
| Running costs NGO |  |  |  |
| --Supplies & Stationery | X | X | X |
| --Communication | X | X | X |
| --Vehicles Purchase/Rental & running costs | X | X | X |
| --Equipment | X | X | X |
| --OTP/TSFP Supplies & Running costs |  | X | X |
| --SC Supplies & Running costs |  | X | X |
| Running costs MoH |  |  |  |
| --OTP/TSFP facility running costs & rental value | X |  |  |
| --OTP/TSFP facility capital costs | X |  |  |
| --OTP/TSFP supplies | X |  |  |
| --SC facility running costs & rental value | X |  |  |
| --SC facility capital costs | X |  |  |
| --SC supplies | X |  |  |

**Supplemental Figure 2. Analytical diagram for adjustment of monthly costs per service type**


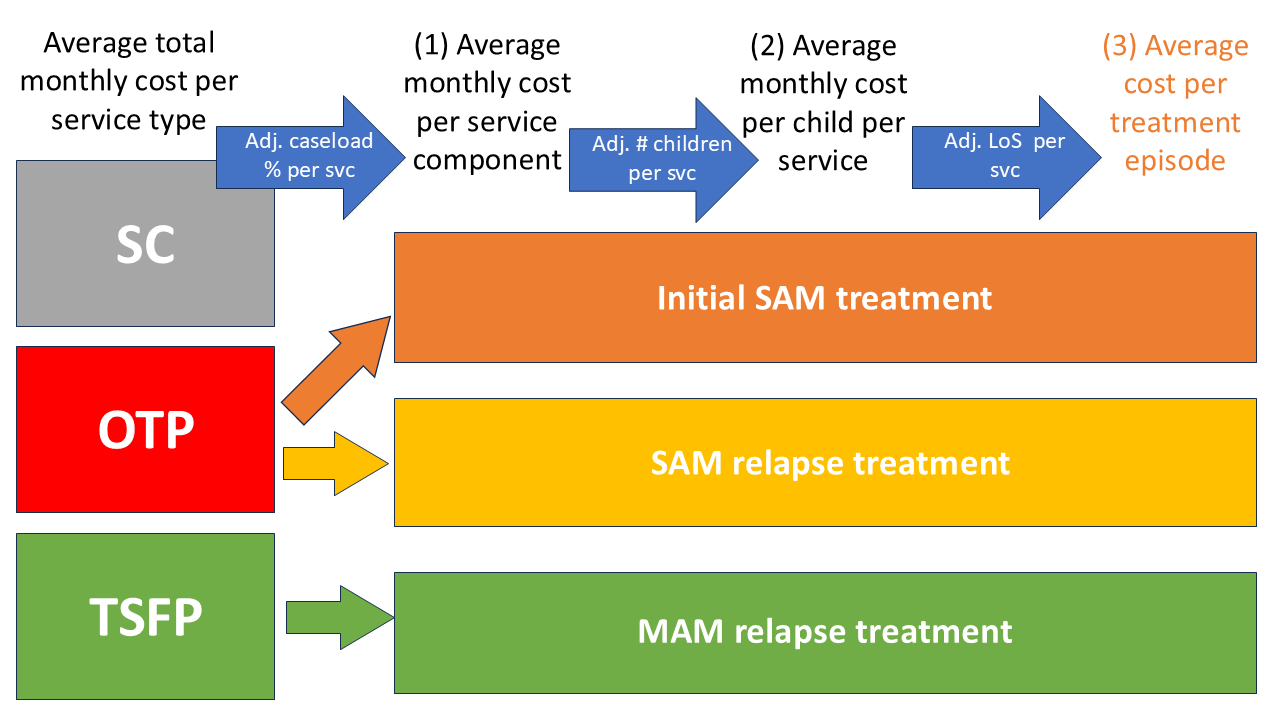


**Supplemental Table 3. Adjustments to monthly cost per service**

|  | Average total monthly cost per service | | | Average monthly cost per service *Adjusted for caseload % per service* | | | Average monthly cost/ child/service  *Adjusted for # children per service* | | | Average cost per treatment episode  *Adjusted for length of stay* | | |
| --- | --- | --- | --- | --- | --- | --- | --- | --- | --- | --- | --- | --- |
|  | Mali | Somalia | SSD | Mali | Somalia | SSD | Mali | Somalia | SSD | Mali | Somalia | SSD |
| Program costs |  |  |  |  |  |  |  |  |  |  |  |  |
| Initial SAM recovery | $9,476◊ | $7,697◊ | $21,843◊ | $5,994 | $5,059 | $6,568 | $91 | $40 | $86 | $163 | $58 | $131 |
| Relapse to SAM∆ | -- | -- | -- | $468 | $144 | $400 | $71 | $50 | $83 | $172 | $47 | $155 |
| Relapse to MAM | $9,269† | $7,697† | $21,843† | $1,029 | $20 | $407 | $69 | $11 | $17 | $111 | $15 | $32 |
| Product costs |  |  |  |  |  |  |  |  |  |  |  |  |
| Initial SAM recovery | $2,988◊ | $4,625◊ | $8,211◊ | $1,395 | $2,369 | $2,278 | $15 | $24 | $30 | $28 | $34 | $47 |
| Relapse to SAM∆ | -- | -- | -- | $119 | $50 | $160 | $18 | $18 | $31 | $43 | $17 | $61 |
| Relapse to MAM | $888† | $7,583† | $13,094† | $88 | $16 | $202 | $5 | $10 | $8 | $7 | $12 | $18 |
| Total costs |  |  |  |  |  |  |  |  |  |  |  |  |
| Initial SAM recovery | $12,464◊ | $12,322◊ | $30,054◊ | $7,389 | $7,698 | $8,846 | $106 | $64 | $116 | $191 | $92 | $178 |
| Relapse to SAM∆ | -- | -- | -- | $587 | $194 | $560 | $89 | $64 | $114 | $215 | $64 | $215 |
| Relapse to MAM | $10,157† | $15,175† | $34,937† | $1,117 | $36 | $609 | $74 | $21 | $25 | $118 | $27 | $50 |

SAM treatment and relapse costs for Mali reflect OTP only; these costs for Somalia and South Sudan reflect both OTP and TSFP services. MAM relapse costs reflect TSFP only for all services. ◊ Costs for OTP only. ∆ OTP costs include both cases of initial SAM recovery and SAM retreatment; therefore, OTP costs include both cases recovering from initial episodes of and relapses to SAM. † Costs for TSFP only.

**Supplemental Table 4. Cost per child recovered from cost analyses presenting complete CMAM program costs**

| **Country** | **Cost per child recovered (US$)** | **SAM or MAM included** | **Description** |
| --- | --- | --- | --- |
| Bangladesh | 165 | SAM | (CHW care; Puett et al., 2013) |
| Ethiopia | 145 | SAM | (CMAM; Tekeste et al., 2012) |
| Mali | 304 | MAM & SAM | (CHW low supervision area; Cichon et al., 2024) |
|  | 325 | MAM & SAM | (CHW medium supervision area; Cichon et al., 2024) |
|  | 312 | MAM & SAM | (CHW high supervision area; Cichon et al., 2024) |
|  | 259 | SAM | (CHW care; Rogers et al., 2018) |
|  | 501 | SAM | (outpatient facility care; Rogers et al., 2018) |
| Pakistan | 382 | SAM | (CHW care; Rogers et al., 2019) |
|  | 363 | SAM | (outpatient facility care; Rogers et al., 2019) |
| **Average all** | 306 |  |  |
| **Median all** | 312 |  |  |
| **Average (SAM only)** | 303 |  |  |
| **Median (SAM only)** | 311 |  |  |

**Studies referenced in Supplemental Table 4:**

Cichon, B., López-Ejeda, N., Mampindu, M. B., Bagayoko, A., Samake, M., & Cuellar, P. C. (2024). Integration of Acute Malnutrition Treatment Into Integrated Community Case Management in Three Districts in Southern Mali: An Economic Evaluation. *Global Health: Science and Practice*.

Puett, C., Sadler, K., Alderman, H., Coates, J., Fiedler, J. L., & Myatt, M. (2013). Cost-effectiveness of the community-based management of severe acute malnutrition by community health workers in southern Bangladesh. Health policy and planning, 28(4), 386-399.

Rogers, E., Martínez, K., Morán, J. L. A., Alé, F. G., Charle, P., Guerrero, S., & Puett, C. (2018). Cost-effectiveness of the treatment of uncomplicated severe acute malnutrition by community health workers compared to treatment provided at an outpatient facility in rural Mali. *Human Resources for Health*, *16*, 1-9.

Rogers, E., Guerrero, S., Kumar, D., Soofi, S., Fazal, S., Martínez, K., Moran, JLA, & Puett, C. (2019). Evaluation of the cost-effectiveness of the treatment of uncomplicated severe acute malnutrition by lady health workers as compared to an outpatient therapeutic feeding programme in Sindh Province, Pakistan. *BMC Public Health*, *19*, 1-11.

Tekeste, A., Wondafrash, M., Azene, G., & Deribe, K. (2012). Cost effectiveness of community-based and in-patient therapeutic feeding programs to treat severe acute malnutrition in Ethiopia. Cost Effectiveness and Resource Allocation, 10, 1-10.
